# Ethylene regulates lateral root formation and auxin transport in *Arabidopsis thaliana*

**DOI:** 10.1111/j.1365-313X.2008.03495.x

**Published:** 2008-05-20

**Authors:** Sangeeta Negi, Maria G Ivanchenko, Gloria K Muday

**Affiliations:** 1 Department of Biology, Wake Forest University, Room 226, Winston Hall, Box 7325Winston-Salem, NC 27109, USA,; 2 Department of Botany and Plant Pathology, 2082 Cordley Hall, Oregon State UniversityCorvallis, OR 97331, USA

**Keywords:** auxin, ethylene, lateral roots, auxin transport, AUX1

## Abstract

Lateral root branching is a genetically defined and environmentally regulated process. Auxin is required for lateral root formation, and mutants that are altered in auxin synthesis, transport or signaling often have lateral root defects. Crosstalk between auxin and ethylene in root elongation has been demonstrated, but interactions between these hormones in the regulation of Arabidopsis lateral root formation are not well characterized. This study utilized Arabidopsis mutants altered in ethylene signaling and synthesis to explore the role of ethylene in lateral root formation. We find that enhanced ethylene synthesis or signaling, through the *eto1-1* and *ctr1-1* mutations, or through the application of 1-aminocyclopropane-1-carboxylic acid (ACC), negatively impacts lateral root formation, and is reversible by treatment with the ethylene antagonist, silver nitrate. In contrast, mutations that block ethylene responses, *etr1-3* and *ein2-5*, enhance root formation and render it insensitive to the effect of ACC, even though these mutants have reduced root elongation at high ACC doses. ACC treatments or the *eto1-1* mutation significantly enhance radiolabeled indole-3-acetic acid (IAA) transport in both the acropetal and the basipetal directions. *ein2-5* and *etr1-3* have less acropetal IAA transport, and transport is no longer regulated by ACC. DR5-GUS reporter expression is also altered by ACC treatment, which is consistent with transport differences. The *aux1-7* mutant, which has a defect in an IAA influx protein, is insensitive to the ethylene inhibition of root formation. *aux1-7* also has ACC-insensitive acropetal and basipetal IAA transport, as well as altered DR5-GUS expression, which is consistent with ethylene altering AUX1-mediated IAA uptake, and thereby blocking lateral root formation.

## Introduction

Plants have an impressive ability to adapt their root development to maximize both reproductive success and survival in harsh environments. Primary roots are formed in the embryo and emerge from the seed during germination. Newly emerged roots are exquisitely sensitive to environmental signals, such as gravity, which acts as a cue to direct root growth towards the moisture and nutrients found below the soil surface (Muday and Rahman, 2007). As roots mature, quiescent cells within their pericycle begin dividing and undergo a precise series of divisions to form a lateral root primordium (Malamy, 2008). Ultimately, the lateral root elongates and undergoes further reiterative branching. The resulting complex root architecture allows maximal nutrient uptake and creates the underground support network that is essential for anchoring the plant.

Auxin has been strongly linked to root growth orientation and lateral root development (Casimiro *et al.*, 2003; Malamy, 2008; Teale *et al.*, 2005). Elevated concentrations of auxin, achieved by auxin application or enhanced synthesis, increase root branching (Boerjan *et al.*, 1995; Celenza *et al.*, 1995; Laskowski *et al.*, 1995; Sitbon *et al.*, 1992; Torrey, 1976). Auxin, with indole-3-acetic acid (IAA) being the predominant naturally occurring auxin, acts at the earliest stages of lateral root primordia initiation, during the activation of the previously quiescent pericycle cells, to begin division (Himanen *et al.*, 2002). Plants with mutations that alter auxin signaling or auxin transport have defects in lateral root initiation or elongation (Malamy, 2008). As auxin transport is a highly regulated process, flexible delivery of auxin to the pericycle cells from which lateral roots initiate is a logical mechanism to tie root development to environmental signaling.

Polar auxin transport occurs via cell-to-cell movement of this hormone (Leyser, 2006). In roots, IAA moves acropetally (from the shoot towards the root apex) through the central cylinder, and moves basipetally (from the root apex toward the base) through the outer root layers (Muday and DeLong, 2001). Auxin transport proteins include IAA efflux carrier complexes, which may include both PIN (pinformed) and ABCB (ATP binding cassette-type B) transporters, also known as MDR/PGP (multidrug resistance-like/P-glycoprotein) proteins (Blakeslee *et al.*, 2005), and IAA influx carriers including AUX1 (auxin insensitive) (Bennett *et al.*, 1996; Yang *et al.*, 2006), and perhaps ABCB4/PGP4/MDR4 (Blakeslee *et al.*, 2007; Cho *et al.*, 2007; Terasaka *et al.*, 2005). These transporters were identified through mutant phenotypes that were linked to auxin transport dependent processes, such as gravitropism, and altered inflorescence and root architecture (Blakeslee *et al.*, 2005; Chen *et al.*, 1998; Lewis *et al.*, 2007; Noh *et al.*, 2001; Okada *et al.*, 1991; Wu *et al.*, 2007), and have been shown to mediate IAA movements across membranes in heterologous expression systems (Blakeslee *et al.*, 2007; Geisler *et al.*, 2005; Petrasek *et al.*, 2006; Terasaka *et al.*, 2005; Yang *et al.*, 2006). In particular, *mdr1/pgp19* has reduced acropetal IAA transport, and forms wild-type numbers of lateral roots, but these lateral roots exhibit reduced elongation (Wu *et al.*, 2007). *aux1* has reduced basipetal and acropetal IAA transport (Rashotte *et al.*, 2001, 2003), and has significant reductions in lateral root formation (Marchant *et al.*, 2002).

Yet, the source of the auxin that reaches the pericycle cells to enhance root formation is still unclear. Inhibition of auxin movement from the shoot into the root reduces lateral root number and is reversible by auxin (Reed *et al.*, 1998). Similarly, quantification of IAA levels in seedlings during germination, and at the earliest stages of root development, suggest that a pulse of IAA moves into the root with timing appropriate to initiate lateral root development (Bhalerao *et al.*, 2002). Additionally, several studies have provided evidence in support of root tip-derived auxin driving root formation. The *stm1* (*shoot meristemless*) mutant, which presumably has reduced IAA synthesis because of missing leaves, still forms lateral roots (Casimiro *et al.*, 2001). An oscillation of DR5-GUS expression has recently been reported in the basal meristem, which might prime pericycle cells for lateral root initiation (De Smet *et al.*, 2007).

Several recent reports have demonstrated crosstalk between auxin and ethylene in Arabidopsis. The hypocotyl and root gravity responses, which are auxin-dependent processes, are inhibited by 1-aminocyclopropane-1-carboxylic acid (ACC), the ethylene precursor (Buer *et al.*, 2006; Muday *et al.*, 2006), as is root waving (Buer *et al.*, 2003). This inhibition requires ETR1 and EIN2: two well-characterized ethylene signaling molecules (Buer *et al.*, 2006; Muday *et al.*, 2006). Root elongation is synergistically inhibited by IAA and ACC (Růžička *et al.*, 2007; Stepanova *et al.*, 2007; Swarup *et al.*, 2007). The inhibition of root elongation by ACC is lost in the *aux1* mutant (Rahman *et al.*, 2001; Růžička *et al.*, 2007), which is consistent with AUX1's central role in the crosstalk between these hormones. Additionally, *arf19*, which has altered auxin-induced gene expression, is less sensitive to the effect of ethylene on root elongation (Li *et al.*, 2006).

One mechanism for the synergy between auxin and ethylene is the positive regulation of auxin synthesis by ethylene (Stepanova *et al.*, 2005, 2007). Another interaction may be through regulation of auxin transport. In some species, ethylene inhibits polar IAA transport in shoot tissues (Morgan and Gausman, 1966; Suttle, 1988), and in roots (Prayitno *et al.*, 2006). Lateral IAA transport in response to gravity is inhibited in both shoots (Burg and Burg, 1966) and gravity-stimulated corn roots (Lee *et al.*, 1990), suggesting that the ethylene-mediated inhibition of auxin transport may regulate the gravity response. Consistent with these transport studies, ACC reduces gravitropism in Arabidopsis roots (Buer *et al.*, 2006) and hypocotyls (Muday *et al.*, 2006) through EIN2- and ETR-dependent mechanisms.

Few reports in the literature have used the array of mutants altered in ethylene signaling or synthesis (Alonso and Stepanova, 2004) to examine the role of ethylene in lateral root formation. The *etr1* and *Neverripe* (*Nr*) mutants of Arabidopsis and tomato, respectively, have reduced ethylene response resulting from the dominant-negative versions of membrane ethylene receptors described in Bleecker *et al.* (1998) and O’Malley *et al.* (2005). The *Nr* tomato mutant has increased underground root mass, consistent with enhanced lateral root formation, but the formation of lateral roots on young *Nr* seedlings was not examined, although alterations in adventitious root formation were reported (Clark *et al.*, 1999). The *ein2* mutant is also ethylene insensitive, but the biochemical function of EIN2 has not been demonstrated (Alonso and Stepanova, 2004). The constitutive triple-response *ctr1* mutant has enhanced ethylene signaling caused by a defect in a gene with sequence similarity to the catalytic domain of the Raf protein kinase (Kieber *et al.*, 1993). The ethylene overproduction mutant, *eto1-3*, has a defect in an ACC synthase gene that stabilizes the protein, thereby enhancing ethylene synthesis (Chae and Kieber, 2005; Guzman and Ecker, 1990). The *polaris* (*pls*) mutant has hallmarks of enhanced ethylene signaling and has reduced lateral root formation (Chilley *et al.*, 2006). The *pls* mutant has reduced IAA transport in the inflorescence and reduced IAA accumulation in roots, consistent with a linkage between ethylene and auxin transport, yet IAA transport and lateral root phenotypes of other well-characterized Arabidopsis ethylene mutants were not described in this or in other reports (Chilley *et al.*, 2006).

This study examined the process of lateral root formation in Arabidopsis seedlings, and tested the hypothesis that root branching is controlled by crosstalk between the plant hormones auxin and ethylene. We examined this question by taking advantage of the diversity of well-characterized mutants that have altered ethylene synthesis and signaling, and by treatments that alter the levels of ethylene in seedlings. We tested the hypothesis that ethylene alters rooting by modulation of polar IAA transport, and have explored the role of the AUX1 protein in mediating the crosstalk between auxin and ethylene in the control of lateral root formation.

## Results

### Lateral root numbers are altered in ethylene-signaling mutants

**Figure 1:**
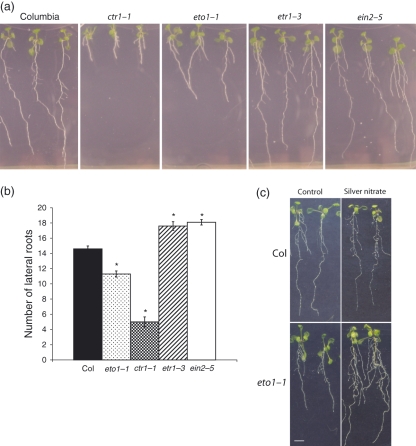
The root branching patterns of Arabidopsis are influenced by mutations that alter ethylene signaling and synthesis. (a) Roots 10 days after sowing are shown. (b) The number of emerged lateral roots in each genotype at 10 days after sowing. The average and SE of 30 seedlings is reported. (c) Images of roots that were grown for 5 days after sowing, before being transferred to control media or media containing 10 μm silver nitrate, and photographed after five additional days of growth. *P* < 0.0005 by a Student’s *t*-test, as compared with untreated Col. Scale bars: 5 mm.

**Figure 2:**
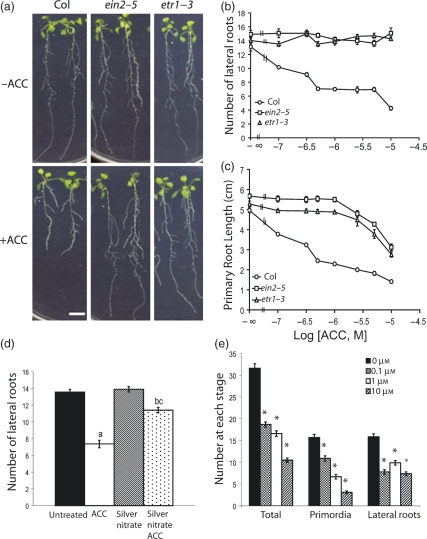
1-Aminocyclopropane-1-carboxylic acid (ACC) reduces root initiation in Col, but not in ethylene-insensitive mutants. Seedlings were grown on control media for 5 days and were then transferred to 1 μm ACC or the indicated concentration, or 10 μm silver nitrate, for five additional days. (a) Lateral root phenotypes are shown with a scale bar of 5 mm. (b and c) The effects of a range of ACC concentrations on the number of lateral roots or the root length were determined, with the average and SE of 30 seedlings reported here (d) The average number of lateral roots and SE of 30 Col seedlings for each treatment are shown with significant differences (*P* < 0.01) compared with untreated seedlings, indicated by the letter ‘a’, or with and without silver treatment, indicated by ‘b’, and silver treatment with or without ACC, indicated by ‘c’ (e) The number of lateral root early primordia, emerged lateral roots, and the combined totals were determined by CYCB1;1:GUS expression. The average and SE of 20 seedlings are reported, and significant differences between treatments and within stages were determined by a Student’s *t*-test, *P*< 0.001.

We examined the role of ethylene in root formation using a genetic approach to alter ethylene concentrations and responsiveness. Root formation in *eto1-1*, an ethylene-overproducing mutant, and *ctr1-1*, a constitutive ethylene-signaling mutant, are compared with wild type in Figure 1(a). Both mutants show a decrease in the number of emerged lateral roots (Figure 1b), along with the well-characterized reduction in primary root elongation (Alonso and Stepanova, 2004). The ethylene-insensitive mutants *ein2-5* and *etr1-3* have increased numbers of lateral roots (Figure 1a,b). In all cases, the number of lateral roots is significantly different from the Columbia parental line, as judged by a Student’s *t*-test. The numbers of lateral roots change in proportion with the overall length of these roots, which is consistent with connections between the roles of ethylene in the elongation of primary roots and in the formation of lateral roots. We also used the inhibitor of ethylene signaling, silver nitrate, to reverse the phenotype of *eto1-1*, as shown in Figure 1(c). In response to silver nitrate treatment, root elongation and branching are enhanced in *eto1-1*, whereas there are only slight, but not statistically significant, enhancements in root branching in wild-type plants (Figure 2d), consistent with ethylene being at limiting concentrations in wild-type seedlings grown in unsealed Petri dishes.

### ACC inhibits both the initiation and the elongation of lateral roots

To raise the levels of ethylene, we treated seedlings with ACC, a precursor of ethylene, and examined lateral root branching in Columbia, *etr1-3*, and *ein2-5* seedlings in the presence and absence of 1 μm ACC, as shown in Figure 2a. Columbia seedlings show both reduced root elongation and reduced lateral root numbers, which phenocopy the *eto1-1* and *ctr1-1* mutations. In contrast, the *etr1-3* and *ein2-5* seedlings show little change in elongation or in lateral root numbers. There is an ACC dose-dependent decrease in lateral root number in wild-type seedlings, which is completely absent in *ein2-5* and *etr1-3* seedlings (Figure 2b). Elongation of primary roots decreases with increasing ACC doses in wild type, but requires higher doses of ACC in *etr1-3* and *ein2-5* to inhibit growth (Figure 2c). In Columbia, the density of lateral roots at several ACC concentrations is constant over a 100-fold range of ACC concentrations, whereas the density of lateral roots for *etr1-3* and *ein2-5* increases, suggesting greater insensitivity to ACC in lateral root numbers than in elongation in these mutants (Figure S1). These results are consistent with ethylene negatively regulating lateral root numbers in an ETR1- and EIN2-dependent fashion. The reduced root branching by ACC is reversible by treatment with the ethylene antagonist silver nitrate (Figure 2d).

The accompanying article contains a detailed analysis showing that ACC affects lateral root initiation in a developmental status or position-specific manner (Ivanchenko *et al.*, 2008). Therefore, we examined the formation of lateral roots on the primary root formed prior to or after transfer to ACC-containing media, as shown in Figure S2. The effect of ACC is constrained to lateral roots formed along the primary root that elongated after transfer to ACC-containing media in both our conditions and in those reported by Ivanchenko *et al.* (2008).

To determine if the negative effect of ethylene on root branching acts through a reduction in root initiation and/or elongation, we used a CYCB1;1:GUS transgenic line that marks dividing cells in the initial stages of lateral root primordia formation (DiDonato *et al.*, 2004). The effect of three concentrations of ACC on primordia and lateral roots is shown in Figure 2(e). With increasing concentrations of ACC, there was a decrease in the number of primordia and elongated lateral roots. These results indicate that ethylene inhibits lateral root formation through the inhibition of primordia formation and the subsequent progression of primordia into elongated lateral roots. Ivanchenko *et al.* (2008) report a similar finding when quantifying primordia in cleared roots.

### Ethylene positively regulates IAA transport

**Figure 3:**
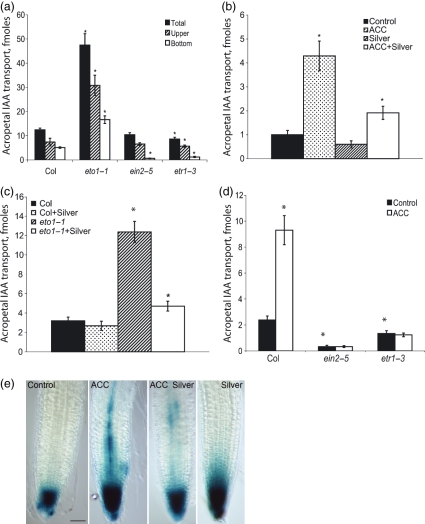
Acropetal indole-3-acetic acid (IAA) transport is positively correlated with ethylene levels and responses. Seedlings were grown on control media for 5 days, and were then transferred to different treatments for 24 h. The average and SE of 30 seedlings are reported in all panels, and statistical analysis was performed using the Student’s *t-*test with significant differences relative to untreated Col indicated; *P < 0.05. (b–d) Tritiated IAA transport was quantified in the apical 5 mm of the root tip. (a) Acropetal transport in several genotypes are compared measuring the radioactivity in three 5-mm segments: upper (near the root shoot junction), middle and tip (b) Col seedlings were treated with 1 μm 1-aminocyclopropane-1-carboxylic acid (ACC) and/or 10 μm silver nitrate (c) Col and *eto1-1* seedlings were treated with 10 μm silver nitrate (d) Col, *ein2-5* and *etr1-3* seedlings were treated with 1 μm ACC (e) DR5:GUS expression in roots treated with 1 μm ACC and/or 10 μm silver nitrate. Scale bar: 40 μm.

We hypothesized that ethylene might reduce lateral root formation by inhibiting acropetal IAA transport. Acropetal IAA transport was measured in intact living seedlings of Col, *eto1-1*, *ein2-5* and *etr1-*3. As there are significant differences in the length of *eto1-1* roots, we quantified the level of tritiated IAA moving from the site of application at the root–shoot junction into the upper and middle sections, matched in position relative to the site of IAA application for all genotypes, and in the root tip, which is found at different lengths from the site of application for *eto1-1*. In all sections, *eto1-1* has enhanced acropetal IAA transport, whereas both *ein2-5* and *etr1-3* showed reduced acropetal auxin transport, particularly in the root-tip segments relative to Col (Figure 3a). These results suggest the opposite of our initial hypothesis, and are consistent with ethylene positively regulating acropetal IAA transport.

Columbia roots were also treated with ACC and silver nitrate, and the effect on acropetal IAA transport was determined, as shown in Figure 3(b). ACC treatment enhanced acropetal auxin transport in Columbia seedlings, and silver nitrate reduced the ability of ACC to enhance IAA transport in the wild type (Figure 3b), and in response to the elevated ethylene synthesis in the *eto1-1* mutant (Figure 3c). Finally, we examined the effect of ACC on acropetal IAA transport in the ethylene-insensitive *ein2-5* and *etr1-3* mutants, and found that both of these genotypes are insensitive to the stimulatory effect of ACC on acropetal IAA transport, as shown in Figure 3(d).

A second method was used to indirectly examine auxin distribution in roots in the presence of altered ethylene levels. The expression of the DR5:GUS reporter in transgenic Columbia seedlings in the presence of ACC, silver nitrate and these molecules in combination is shown in Figure 3(e). There is enhanced expression of this auxin-inducible construct in the tissues of the central cylinder of the root with ACC treatment, which is reversed by silver treatment. As acropetal IAA transport occurs in the central cylinder, these results are also consistent with ethylene positively regulating acropetal IAA transport.

**Figure 4:**
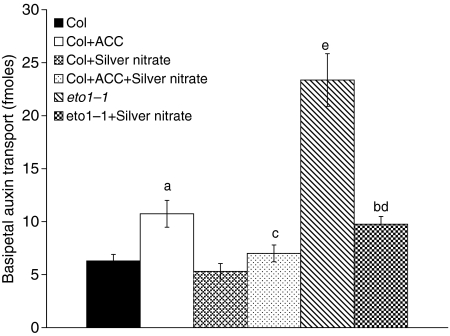
Basipetal indole-3-acetic acid (IAA) transport is increased with 1-aminocyclopropane-1-carboxylic acid (ACC) treatment and in *eto1-1* seedlings Five-day-old Col or *eto1-1* seedlings were treated with 1 μm ACC, 10 μm silver nitrate or both, and basipetal IAA transport was quantified after 24 h of treatment. The average and SE of 30 seedlings are reported, and statistical analysis was performed using the Student’s *t-*test, with all values being significant with *P* < 0.0001, with the following comparisons within genotypes: a, with and without ACC; b, with and without silver nitrate; c, with ACC and with and without silver nitrate, and between genotypes; d, untreated Col versus silver nitrate treated *eto1*; and e, untreated Col versus *eto1-1.*

We also examined the effect of these treatments on basipetal IAA transport. ACC and the *eto1-1* mutation enhance basipetal IAA transport, as shown in Figure 4. Silver nitrate reduced transport and prevented the enhancement by elevated ethylene levels in both ACC-treated controls and *eto1-1*. These results indicate that ethylene has similar stimulatory effects on acropetal and basipetal IAA transport.

**Figure 5:**
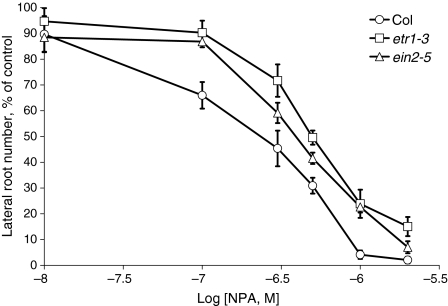
Ethylene-insensitive mutants are less sensitive to naphthylphthalamic acid (NPA). Col, *ein2-5* and *etr1-3* seedlings were grown on control media, and after 5 days were transferred to media containing the indicated doses of NPA. After 5 days of growth in the presence of NPA, the number of emerged lateral roots was quantified. Data are presented as a percentage of lateral roots in the untreated control. The average and SE of 20 seedlings are reported.

The effect of the IAA efflux inhibitor naphthylphthalamic acid (NPA) on root formation was examined in Col, *ein2-5* and *etr1-3* mutants, as shown in Figure 5. Consistent with the altered regulation of acropetal IAA transport in these mutant genotypes, there is reduced sensitivity to the inhibition of lateral root formation by NPA compared with Columbia seedlings.

### The negative effect of ethylene on lateral root formation can be reversed by auxin

**Figure 6:**
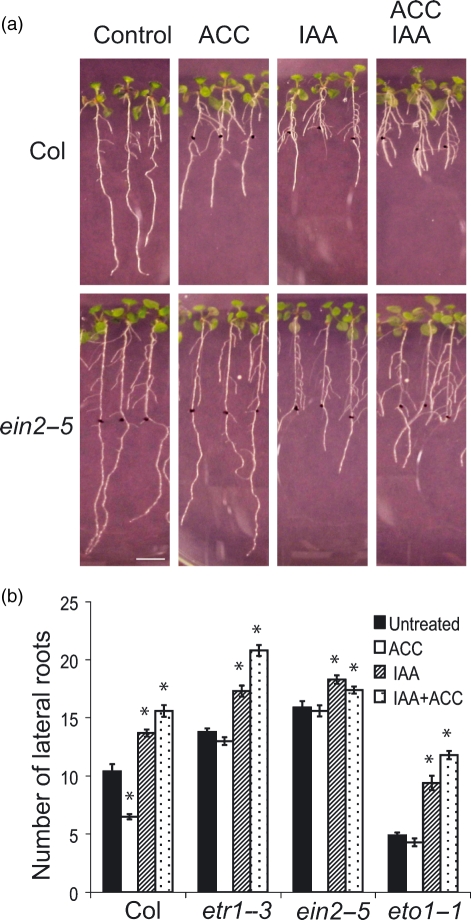
Exogenous indole-3-acetic acid (IAA) can reverse the negative effect of 1-aminocyclopropane-1-carboxylic acid (ACC) on lateral root formation. Col, *ein2-5* and *eto1-1* seedlings were grown on control media, and after 5 days were transferred to media containing 1 μm ACC, 1 μm IAA or both. (a) At the time of transfer, the plate was marked with a dot at the position of the root tip. After 5 days of growth in the presence of the indicated treatments, images of roots were captured. (b) The number of lateral roots was quantified. The average and SE of 30 seedlings and significant differences within genotypes are reported; *P< 0.001.

One model that considers the negative effect of ethylene on lateral root formation and the positive effect on IAA transport is that ethylene might block the IAA signaling pathway required for the positive regulation of root formation. To test this possibility, we treated seedlings with either ACC or IAA, or both, and the effect on root formation is shown in Figure 6. The dots on each photograph illustrate the position of the root tip at the time of transfer. In wild-type roots, ACC reduces lateral root formation, with more profound effects on regions of the root that form after transfer to ACC-containing media. In contrast, IAA increases root formation with more profound effects in the regions that were formed prior to exposure to IAA. Combination treatments indicate that the positive IAA effect dominates over the negative regulation of root formation by ethylene. These results are inconsistent with the model that ethylene renders roots unable to respond to IAA.

The effect of IAA and ACC on root formation was also examined in *ein2-5*, *etr1-3* and *eto1-1*, as shown in Figure 6. In all genotypes, IAA enhanced lateral root formation, whereas ACC alone had no effect in any of these genotypes. In both *etr1-3* and *eto1-1*, simultaneous treatment with ACC and IAA amplified the effect of IAA alone, whereas in *ein2-5*, ACC had no additional effect when added in concert with IAA. Ethylene does not prevent the effect of IAA in any of these genotypes. The effect of a range of IAA concentrations on lateral root formation in several genotypes is compared in Figure S3. *etr1-1* and *ein2-5* have a greater number of lateral roots in the absence of IAA and show similar enhancement of lateral root formation at all IAA doses, consistent with the ethylene sensitivity not being required for IAA-enhanced lateral root formation.

### aux1-7 is resistant to the negative effect of ethylene on lateral root formation

**Figure 7:**
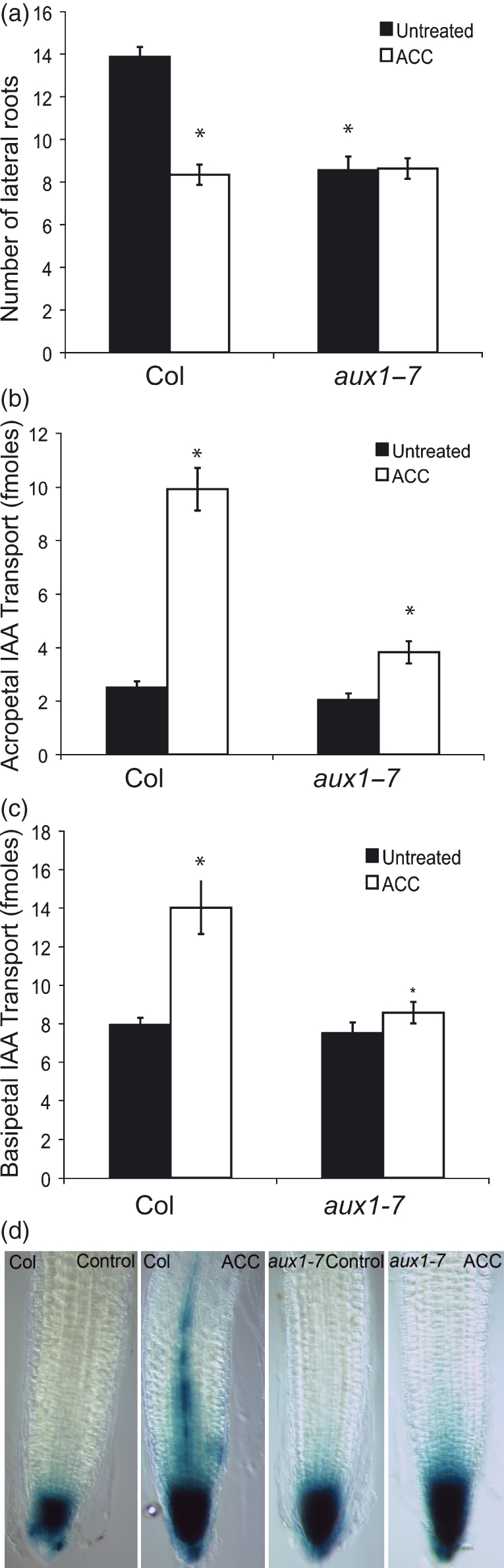
Lateral root formation and indole-3-acetic acid (IAA) transport are less ethylene-sensitive in *aux1-7* (a) Col and *aux1-7* were transferred to media containing 1 μm 1-aminocyclopropane-1-carboxylic acid (ACC) at 5 days after sowing, and the number of emerged lateral roots was quantified after five additional days (b) The effect of ACC on acropetal IAA transport in Col and *aux1-7* are compared (c) The effect of 1 μm ACC on basipetal transport in *aux1-7* and Col are compared (d) *DR5:*GUS expression is shown in Col and *aux1-7* in the presence and absence of 1 μm ACC; the scale bar is 40 μm. For all experiments, the average and SE of 20–30 seedlings, and significant differences, are reported for comparisons between untreated genotypes or for treatment within genotypes; *P< 0.001.

We developed a second hypothesis for the interconnection between the effects of ACC in reducing lateral root formation and enhancing acropetal IAA transport. When acropetal IAA transport is enhanced by elevated ethylene synthesis or signaling, perhaps this limits the quantity of auxin that can leave the polar transport stream to be taken up into pericycle cells to stimulate root formation. A likely candidate to facilitate the IAA unloading needed for lateral root initiation is AUX1, as expression of the *AUX1* gene is one of the earliest markers in lateral root initiation (Malamy, 2005; Marchant *et al.*, 2002). We examined the effect of ACC on root formation in *aux1-7*, as shown in Figure 7(a). We find that the inhibitory effect of ACC is lost in *aux1-7*, consistent with the ACC treatment acting on AUX1 to reduce root formation. We used two auxin influx inhibitors to phenocopy *aux1*, and find that both 1-naphthoxyacetic acid and 3-chloro-4-hydroxyphenylacetic acid (Parry *et al.*, 2001) prevent the effect of ACC on lateral root inhibition (Figure S4a), indicating a central role for IAA influx in this process.

We also examined acropetal and basipetal IAA transport in *aux1-7*. Like lateral root formation, there is a significant reduction in the ability of ACC to regulate acropetal IAA transport in *aux1-7* (Figure 7b). Although there is still a significant stimulation of transport by ACC in *aux1-7* relative to untreated *aux1-7*, there is a significant difference between ACC-treated genotypes. We find a similar pattern in basipetal IAA transport assays, in which ACC-stimulated transport is significantly reduced in the *aux1-7* mutant (Figure 7c). These results show that AUX1 is required for most of the ethylene-enhanced IAA transport in both polarities. We were surprised that there was little difference in basipetal transport between the untreated wild type and *aux1-7*, as we had previously observed these differences (Rashotte *et al.*, 2001, 2003). As our previous experiments were performed with plates that were wrapped, and were likely to have had elevated ethylene levels, we wondered whether the parafilm sealing of plates was sufficient to enhance transport relative to unwrapped plates in wild type, but not in *aux1-7*. Wrapped plates have substantially higher levels of ethylene (Buer *et al.*, 2003) and, as shown in Figure S4(b), under these conditions we find significant differences in basipetal transport between Col and *aux1-*7, consistent with previous reports. Similar to the transport differences, *DR5:GUS* gene expression is similar in Col and *aux1-7* in untreated plants, but more profound differences are detected in the presence of ACC (Figure 7d).

## Discussion

This study tested the hypothesis that Arabidopsis lateral root development is controlled by crosstalk between the plant hormones auxin and ethylene. Elevated levels of ethylene, either achieved through the application of ACC or by enhanced ethylene synthesis in *eto1*, reduced the numbers of lateral roots and primary root elongation growth. This effect is reversible by treatment with silver nitrate, an ethylene antagonist. Similarly, the constitutive ethylene signaling *ctr1* mutant shows reduced numbers of lateral roots. The effect of enhanced ethylene synthesis and signaling is at the level of root initiation, as quantification of lateral root primordia with a *cycl1At*-GUS reporter line demonstrates reductions in the numbers of all stages of lateral roots, with the most profound effect on early stages of lateral root formation. In contrast, mutants with reduced ethylene sensitivity, such as *ein2* and *etr1*, show enhanced lateral root formation. We find an ACC dose-dependent decrease in root branching in Columbia, which is absent in *etr1* and *ein2*.

These mutations or treatments that alter ethylene signaling or synthesis affect root elongation and lateral root formation in parallel. Calculations of lateral root density along the primary root are roughly constant with increasing ACC concentration in Col. The one exception is when *etr1* and *ein2* roots are treated with high doses of ACC, where the inhibition of root elongation is more profound, and so the lateral root density increases. This finding is consistent with a strong requirement for ETR and EIN2 function for ethylene regulation of root formation. Another study showed that the effect of ACC on lateral root density is relatively constant over a 25-fold ACC concentration range (Ivanchenko *et al.*, 2008). When the lateral root initiations are quantified for 100 cells, there are significant decreases in lateral root frequency at 0.2 μm ACC and above (Ivanchenko *et al.*, 2008), suggesting that the examination of lateral root density alone does not provide accurate information on the cellular frequency of root formation.

The effect of ethylene on root formation is enhanced in regions nearer the growing tip that are formed after exposure to ACC (Figure S2). Ivanchenko *et al.* (2008) quantified the number of lateral root initiation events in the mature root formed before transfer, and in the root formed after transfer to ACC. They found that the number of roots in the mature region is unchanged by ACC treatment, whereas there are fewer lateral roots initiated in the newly formed region at doses of ACC greater than 0.2 μm. Here, we have consistently reported on the total number of lateral roots formed in different treatments or mutants. This quantification does not change the conclusions about the effect of each treatment, although it may under-represent the magnitude of the negative effect of ethylene, as treated roots have greater effects in one region than in the whole root.

Several recent studies demonstrate crosstalk between ethylene and auxin in root elongation (Rahman *et al.*, 2001; Růžička *et al.*, 2007; Stepanova *et al.*, 2007; Swarup *et al.*, 2007). These reports suggested that ethylene positively regulates IAA transport using auxin-responsive reporter constructs, which provide an indirect method of observation of IAA transport. These reporters provide information on the capacity to respond to IAA, as well as IAA concentrations (Růžička *et al.*, 2007; Stepanova *et al.*, 2007; Swarup *et al.*, 2007). We directly tested the possibility that IAA transport was altered by ethylene signaling and synthesis by measuring the movement of radiolabeled IAA in whole living Arabidopsis seedlings, to understand the regulation of IAA transport by ethylene. Disruption of IAA transport with efflux inhibitors blocks lateral root initiation and elongation (Casimiro *et al.*, 2001; Reed *et al.*, 1998), so we hypothesized that ACC treatment would reduce IAA transport.

We find that rather than decreasing IAA transport, ACC treatment or the *eto1* mutation led to a 4- to 5-fold enhancement of root acropetal IAA transport relative to untreated Col roots, and to a twofold enhancement of basipetal IAA transport. Mutations that reduce ethylene responsiveness, *etr1* and *ein2*, reduce acropetal IAA transport and render it insensitive to the effects of ethylene. Finally, silver nitrate significantly reduces the enhanced acropetal IAA transport in ACC-treated seedlings and in the *eto1* mutant. DR5-GUS expression was examined as a secondary and indirect measure of changes in IAA distribution in response to ACC. In particular, the expression of DR5-GUS in the vascular tissues of the root, which are likely to mediate acropetal IAA transport, is enhanced in seedlings treated with 1 μm ACC, and silver nitrate prevents this enhancement. This finding is consistent with ethylene enhanced acropetal IAA transport, and our images resemble similar reports of changes in DR5-GUS in response to 10 μm ACC (Stepanova *et al.*, 2007).

The positive effect of ethylene on auxin transport reported here was surprising, for several reasons. In some species, ethylene inhibits polar IAA transport in shoot tissues (Morgan and Gausman, 1966; Suttle, 1988). A recent report indicates that in roots of *Medicago*, ethylene reduces acropetal IAA transport (Prayitno *et al.*, 2006). As the *Medicago* roots were grown on nitrogen- and sucrose-free media under different light conditions, the resolution of whether this difference in the effect of ethylene on auxin transport are caused by species or growth-condition differences awaits additional experimentation.

Our initial hypothesis that ethylene-mediated reductions in lateral root formation would be accompanied by reduced IAA transport was not supported by the data. We therefore developed two alternative models to explain the enhanced polar IAA transport coupled with reduced branching. In the first model, we hypothesized that ethylene might reduce the ability of roots to respond to IAA. We tested this model by treating roots with ACC and IAA simultaneously, and we found that the Col and mutant lines continued to positively respond to IAA, even in the presence of ACC, although the majority of IAA-induced root formation occurs in the older parts of the root. Therefore, ACC does not uniformly block the auxin responsiveness of root formation.

As ethylene does not prevent the response to auxin, then the role of ethylene in controlling root branching is likely to alter the availability of auxin. As exogenous auxin enhances lateral root initiation at physiological, and well-above physiological, concentrations (Poupart *et al.*, 2005), perhaps the enhanced transport of auxin through the root limits the auxin that remains in the root to locally stimulate lateral root formation. Therefore, we developed the alternative hypothesis that the ethylene enhancement of polar IAA transport might alter the quantity of IAA that unloads into the pericycle cells to drive lateral root formation. AUX1 is the best-characterized influx carrier protein in Arabidopsis, the *aux1* mutant has defects in lateral root development and *AUX1* is expressed in developing lateral root primordia (Marchant *et al.*, 2002), suggesting that AUX1 is a likely target to mediate IAA influx into developing lateral root primordia to prime lateral root formation.

We therefore examined the effect of ACC on lateral root formation, and acropetal and basipetal IAA transport, in the *aux1* mutant. In *aux1-7*, lateral root formation is reduced, the effect of ACC on root formation is lost, and acropetal and basipetal IAA transport are less responsive to ACC treatment than in wild type. A mechanism by which transport is enhanced in the wild type may be through increased *AUX1* expression. This prediction is consistent with the previous report that ethylene enhances the expression of *AUX1* reporters after ACC treatment (Růžička *et al.*, 2007). Together, these results are consistent with ethylene acting through AUX1 protein to enhance long-distance polar IAA transport, and to alter IAA availability for lateral root formation, by reducing levels in tissues stimulated by IAA to form lateral roots. As AUX1 has roles in acropetal and basipetal transport in primary roots, and may facilitate IAA transport into developing lateral roots, these experiments cannot yet dissect which transport stream is tied to the reduction in lateral root formation by ethylene. Furthermore, elucidation of the mechanism by which auxin moves into the pericycle cells, from which lateral roots are initiated, awaits additional experimentation.

Several reports have uncovered mechanisms for the synergistic negative regulation of root elongation by auxin and ethylene, including crosstalk between the synthesis of auxin and ethylene. Auxin positively regulates ethylene biosynthesis (Woeste *et al.*, 1999) through auxin-inducible expression of ACC synthase, which catalyzes the rate-limiting step in ethylene synthesis (Abel *et al.*, 1995; Yamagami *et al.*, 2003). Ethylene enhances auxin synthesis in the root tip through WEI2 and WEI7 (Růžička *et al.*, 2007; Stepanova *et al.*, 2005, 2007; Swarup *et al.*, 2007). DR5-GUS and DR5-GFP expression have been shown to be enhanced in the epidermis (Růžička *et al.*, 2007), whereas other reports indicate that DR5-GUS or IAA2-GUS are increased by elevated ethylene in the epidermis and central cylinder (Stepanova *et al.*, 2007; Swarup *et al.*, 2007). These reports are suggestive of ethylene-enhanced IAA transport in cells of the epidermis and central cylinder, which mediate basipetal and acropetal IAA transport, respectively. Finally, *aux1* is reduced in its response to the effect of ACC on root elongation (Rahman *et al.*, 2001; Stepanova *et al.*, 2007; Swarup *et al.*, 2007), which is consistent with the defective IAA transport of *aux1*. Together, these mechanisms for interactions between IAA and ethylene logically explain the experimental observations for the synergistic inhibition of primary root growth.

The mechanism for the IAA and ethylene antagonism during lateral root formation is more complex to dissect than the synergistic action of these two hormones on root elongation. Ethylene-enhanced IAA synthesis (Stepanova *et al.*, 2007; Swarup *et al.*, 2007) and IAA transport (this work) would be predicted to positively regulate lateral root formation, as elevations in endogenous and exogenous auxin stimulate lateral root development. An alternative possibility is that ethylene could prevent the response of pericycle cells to IAA, but the inability of ethylene to block IAA-enhanced root formation argues against this possibility.

We developed a more complex transport model, in which the IAA that was required to drive lateral root formation might reach the pericycle cells through a two-step process, in which polar transport and unloading into the pericycle cells may be unlinked. As ACC enhances both acropetal and basipetal IAA transport, we cannot resolve the stream of auxin that is tied to this process, and it is possible that both flows ultimately raise IAA levels in the basal meristematic region that has important signaling roles in lateral root formation (De Smet *et al.*, 2007), either directly or through a reflux loop that has been identified through genetic experiments (Blilou *et al.*, 2005; Lewis *et al.*, 2007).

We tested the possibility that AUX1 might be a critical player in controlling this balance of polar transport and IAA unloading into the pericycle in response to elevated ethylene levels. The absence of ethylene-enhanced polar transport and ethylene-inhibited lateral root formation in this mutant, and in influx inhibitor-treated seedlings, is consistent with the central importance of IAA uptake in lateral root development. These results are also consistent with previous reports that AUX1 plays a critical role in the interface between ethylene levels and the regulation of root growth and development (Stepanova *et al.*, 2007; Swarup *et al.*, 2007). As AUX1 is expressed in the root tip, in developing lateral roots and in the shoot meristem (Marchant *et al.*, 2002), it is not possible to determine which of these sites control the movement of auxin needed to inhibit lateral root formation in response to ethylene. The reduced acropetal transport in *aux1* may be caused by the altered loading of auxin from the leaves and cotyledons (Marchant *et al.*, 2002) into the acropetal IAA transport stream. Even though the role of AUX1 in mediating IAA transport is complex, AUX1 is clearly tied to ethylene-regulated root development.

Together, these experiments demonstrate a profound effect of ethylene on lateral root initiation in Arabidopsis, which works through the well-characterized ethylene signaling pathway. The effect is likely to be a result of crosstalk with auxin signaling and auxin transport pathways. These changes in lateral root initiation lead to new patterns of root architecture that may have adaptive significance under conditions in which ethylene is elevated.

## Experimental procedures

### Chemicals

Triton X-100 was purchased from Fisher Scientific (http://www.fisher.co.uk). NPA was purchased from Chem Service (http://www.chemservice.com). MS salts were purchased from Caisson Labs (http://www.caissonlabs.com). [5--^3^H]IAA was purchased from Amersham (25 Ci mmol^−1^; http://www.amersham.co). All other chemicals were acquired from Sigma-Aldrich (http://www.sigmaaldrich.com).

### Plant material and growth conditions

*etr1-3* and *ein2-5* were used previously (Buer *et al.*, 2006). *eto1-1* was provided by Kieber *et al.* (1993), *ctr1-1* and *aux1-7* containing DR5-GUS were provided by Stepanova *et al.* (2007) and *cycl1At*:GUS seeds were provided by DiDonato *et al.* (2004). All seeds were sterilized by incubation for 1 min in 95% ethanol, followed by 5 min in freshly prepared 20% (v/v) bleach plus 0.01% (v/v) Triton X-100, and were then washed with sterile water. The sterilized seeds were sown on control plates: 0.8% (w/v) Type-M agar (A-4800; Sigma-Aldrich), MS nutrients (macro and micro salts, MSP0501; Caisson Labs), vitamins (Murashige and Skoog, 1962), 1.5% (w/v) sucrose, 0.05% (w/v) 2-(*N*-morpholine)-ethanesulphonic acid (MES), with pH adjusted to 6.0. Plated seeds were stratified at 4°C in dark conditions for 2 days to induce even germination. The unsealed plates were placed vertically in racks, and the seedlings were grown under constant fluorescent lights of 100 μmol m^−2^ sec^−1^ at 23°C. For experiments with IAA, plates were kept under yellow filters to prevent IAA degradation (Stasinopoulos and Hangarter, 1989).

### Lateral root quantification

Seedlings were germinated, and at 5 days after sowing were transferred to new media, containing either no additions or the indicated quantities of ACC, silver nitrate, NPA or IAA. The number of lateral roots on the primary root was counted under a dissecting microscope after five additional days of growth; all lateral roots that had emerged from the primary root were counted. In the case of CYC1B:GUS seedlings, lateral root primordia, emerged lateral roots and elongated lateral roots were separately quantified using a dissecting microscope.

### Auxin transport assays

Seedlings were germinated on control media and transplanted to control or treatment plates on the fifth day following sowing. After 24 h of treatment, a 100 nm [^3^H]IAA agar cylinder was applied just below the aligned root shoot junctions, and the seedlings were incubated in the dark in the inverted position, to prevent [^3^H]IAA from diffusing along the root, for 18 h. Root segments were excised and the level of radioactivity quantified in two ways. For the first experiment with multiple genotypes, which have different primary root lengths, the roots were divided into 5-mm upper segments (upper being closest to the shoot), middle segments and a 5-mm lower segment (the root tip). For other assays, the roots were not significantly different from controls, so the apical 5 mm of each root tip was excised. Individual segments from each plant and position were placed in 2.5 ml of scintillation liquid [Scintiverse (TM) BD cocktail; Fisher Scientific) in 3-ml scintillation vials, and radioactivity was measured for 2 min on a Beckman scintillation counter (model LS 6500; Beckman Coulter, http://www.beckmancoulter.com).

Measurement of radioactive basipetal auxin transport was performed using the method of Rashotte *et al.* (2003), using seedlings 5 days after sowing and 18 h after treatment with the indicated compounds.

β-Glucuronidase staining was performed with Columbia or *aux1-7* DR5-GUS (Ulmasov *et al.*, 1997) and *cyc1At*-GUS seedlings according to previously published procedures (Buer and Muday, 2004). Seedlings were treated with 1 μm ACC and/or 10 μm silver nitrate for 24 h, and were then washed with 50 mm sodium phosphate buffer (pH 7.0).

## Supplementary Material

The following supplementary material is available for this article online:

Figure S1 The lateral root density is not changed by 1-aminocyclopropane-1-carboxylic acid (ACC) in the wild type, but is increased in ethylene-insensitive mutants.

Figure S2 1-Aminocyclopropane-1-carboxylic acid (ACC) affects lateral root formation in the regions forming after transfer to ACC-containing media.

Figure S3 Ethylene-insensitive mutants have indole-3-acetic acid (IAA) enhanced lateral root formation in proportion to the effect of IAA in the Columbia wild-type background.

Figure S4 (a) Treatment with IAA influx inhibitors prevents the ACC inhibition of lateral root formation. (b) Wrapping plates in parafilm, like ACC treatment, enhances basipetal IAA transport in Col, but not *aux1-7*.

This material is available as part of the online article from http://www.blackwell-synergy.com.
