# Supplementary figures and images for: Ethylene regulates lateral root formation and auxin transport in Arabidopsis thaliana
Source: Plant J. 2008 May 20;55(2):175–87. doi: 10.1111/j.1365-313X.2008.03495.x (PMC2635504; doi:10.1111/j.1365-313X.2008.03495.x)

Supplemental Figure 1

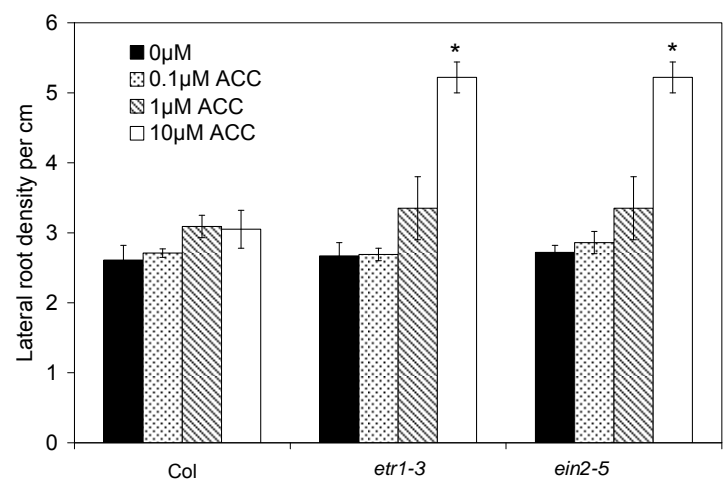

Supplemental Figure 2

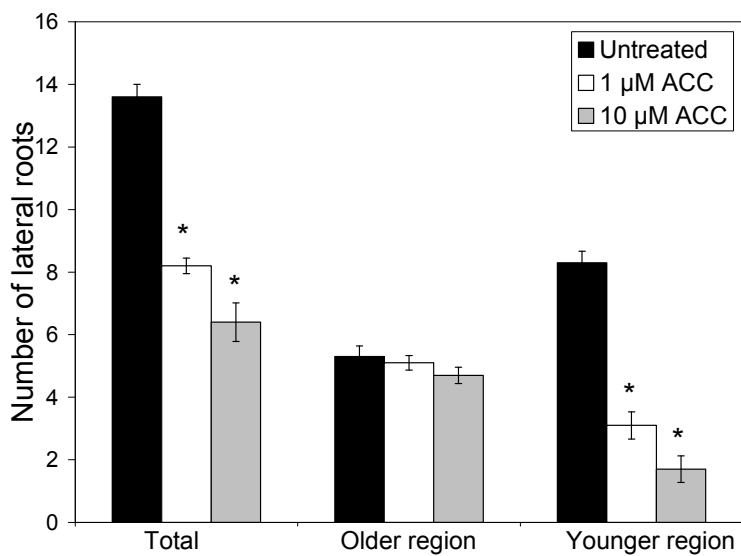

Supplemental Figure 3:

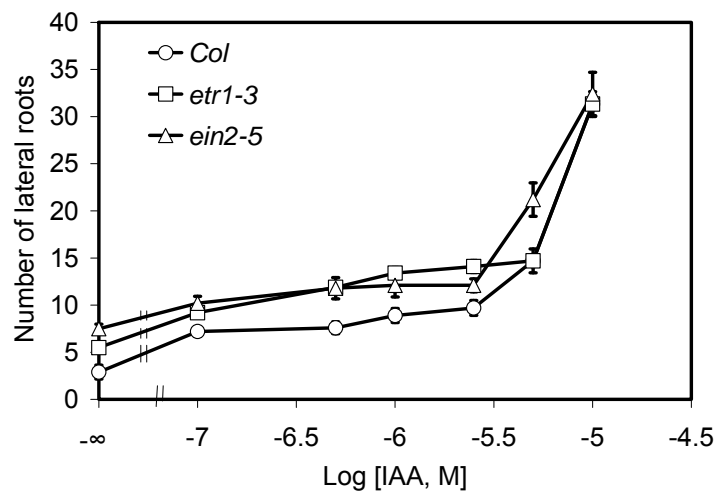

Supplemental Figure 4:

(a)

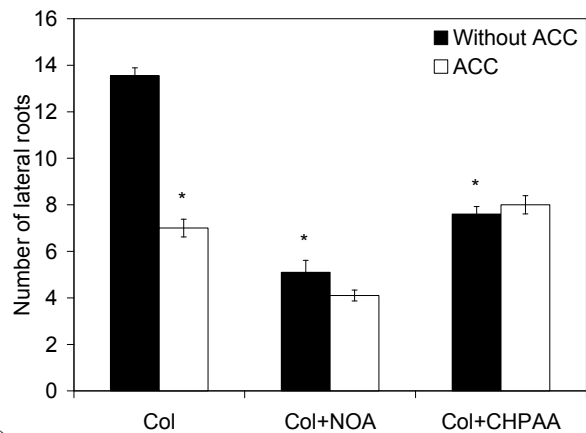

(b)

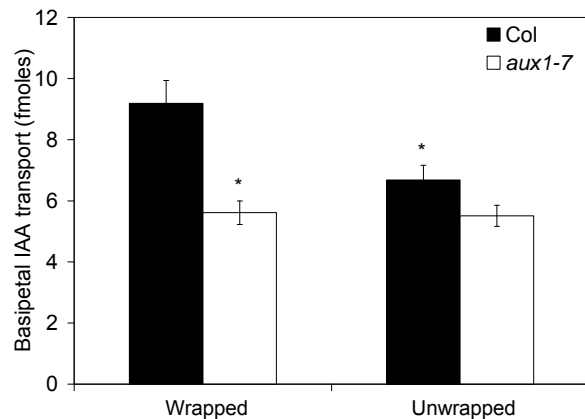

Supplement: Figure S1 [file tpj0055-0175-SD1.pdf]
